# Supplementary material for: Pressure Injury Prediction in Intensive Care Units Using Artificial Intelligence: A Scoping Review
Source: Nurs Rep. 2025 Apr 9;15(4):126. doi: 10.3390/nursrep15040126 (PMC12030323; doi:10.3390/nursrep15040126)
Supplement: Supplementary file 1 [file nursrep-15-00126-s001.zip › Supplementary Material S1.pdf]

Supplementary Material S1: Search strategy in selected databases.

Database (Host)	Search String	Results
CINAHL (EBSCO)	(TI ("ICU" OR "Critical Care" OR "Intensive Care" OR "Critical Illness" OR "Critically Ill Patient*" OR "Intensive Care Unit*") OR AB ("ICU" OR "Critical Care" OR "Intensive Care" OR "Critical Illness" OR "Critically Ill Patient*" OR "Intensive Care Unit*") OR MH ("Critical Care" OR "Intensive Care OR "Intensive Care Units" OR "Critical Illness" OR "Critically Ill Patients")) **AND** (TI ("Machine Learning" OR "Artificial Intelligence" OR "Deep Learning" OR "Supervised Machine Learning" OR "Unsupervised Machine Learning" OR "Neural Networks" OR "Support Vector Machine") OR AB ("Machine Learning" OR "Artificial Intelligence" OR "Deep Learning" OR "Supervised Machine Learning" OR "Unsupervised Machine Learning" OR "Neural Networks" OR "Support Vector Machine") OR MH ("Machine Learning" OR "Artificial Intelligence" OR "Deep Learning" OR "Support Vector Machine" OR "Neural Networks")) **AND** (TI ("Pressure ulcer*" OR Bedsore* OR "Bed Sore*" OR "Decubitus Ulcer*" OR "Pressure Sore*" OR "Pressure Injur*") OR AB ("Pressure ulcer*" OR Bedsore* OR "Bed Sore*" OR "Decubitus Ulcer*" OR "Pressure Sore*" OR "Pressure Injur*") OR MH ("Pressure Ulcer"))	10
MEDLINE (EBSCO)	(AB ("ICU" OR "Critical Care" OR "Intensive Care" OR "Critical Illness" OR "Critically Ill Patient*" OR "Intensive Care Unit" OR TI ("ICU" OR "Critical Care" OR "Intensive Care" OR "Critical Illness" OR "Critically Ill Patient*" OR "Intensive Care Unit") OR MH ("Critical Care" OR "Intensive Care" OR "Critical Illness" OR "Intensive Care Units"))) **AND** (AB ("Machine Learning" OR "Artificial Intelligence" OR "Deep Learning" OR "Supervised Machine Learning" OR "Unsupervised Machine Learning" OR "Neural Networks" OR "Support Vector Machine") OR TI ("Machine Learning" OR "Artificial Intelligence" OR "Deep Learning" OR "Supervised Machine Learning" OR "Unsupervised Machine Learning" OR "Neural Networks" OR "Support Vector Machine") OR MH ("Machine Learning" OR "Artificial Intelligence" OR "Deep Learning" OR "Supervised Machine Learning" OR "Unsupervised Machine Learning" OR "Neural Networks"))) **AND** (AB ("Pressure ulcer*" OR Bedsore* OR "Bed Sore*" OR "Decubitus Ulcer*" OR "Pressure Sore*" OR "Pressure Injur*") OR TI ("Pressure ulcer*" OR Bedsore* OR "Bed Sore*" OR "Decubitus Ulcer*" OR "Pressure Sore*" OR "Pressure Injur*") OR MH ("Pressure Ulcer"))	15
SCOPUS	TITLE-ABS-KEY ("Pressure ulcer*" OR bedsore* OR "Bed Sore*" OR "Decubitus Ulcer*" OR "Pressure Sore*" OR "Pressure Injur*") **AND** TITLE-ABS-KEY ("Machine Learning" OR "Artificial Intelligence" OR "Deep Learning" OR "Supervised Machine Learning" OR "Unsupervised Machine Learning" OR "Neural Networks" OR "Support Vector Machine") **AND** TITLE-ABS-KEY ("ICU" OR "Critical Care" OR "Intensive Care" OR "Critical Illness" OR "Critically Ill Patient*" OR "Intensive Care Unit*")	22
PubMed	(((("ICU"[Title/Abstract] OR "Critical Care"[Title/Abstract] OR "Intensive Care"[Title/Abstract] OR "Critical Illness"[Title/Abstract] OR "Critically Ill Patient*"[Title/Abstract] OR "Intensive Care Unit*"[Title/Abstract] OR ("Critical Care"[Title/Abstract] OR "Intensive Care"[Title/Abstract] OR "Critical Illness"[Title/Abstract] OR "Intensive Care Units"[Title/Abstract]))))) **AND** (("Machine Learning"[Title/Abstract] OR "Artificial Intelligence"[Title/Abstract] OR "Deep Learning"[Title/Abstract] OR "Supervised Machine Learning"[Title/Abstract] OR "Unsupervised Machine Learning"[Title/Abstract] OR "Neural Networks"[Title/Abstract] OR "Support Vector Machine"[Title/Abstract] OR ("Machine Learning" OR "Artificial Intelligence" OR "Deep Learning" OR "Supervised Machine Learning" OR "Unsupervised Machine Learning" OR "Neural Networks, Computer"[MeSH Terms]))) **AND** (("Pressure ulcer*"[Title/Abstract] OR Bedsore*[Title/Abstract] OR "Bed Sore*"[Title/Abstract] OR "Decubitus Ulcer*"[Title/Abstract] OR "Pressure Sore*"[Title/Abstract] OR "Pressure Injur*"[Title/Abstract] OR ("Pressure Ulcer"[MeSH Terms])))	20
Cochrane Library	("Pressure ulcer*" OR Bedsore* OR "Bed Sore*" OR "Decubitus Ulcer*" OR "Pressure Sore*" OR "Pressure Injur*") in **Title Abstract Keyword AND** ("Machine Learning" OR "Artificial Intelligence" OR "Deep Learning" OR "Supervised Machine Learning" OR "Unsupervised Machine Learning" OR "Neural Networks" OR "Support Vector Machine") in **Title Abstract Keyword AND** ("ICU" OR "Critical Care" OR "Intensive Care" OR "Critical Illness" OR "Critically Ill Patient*" OR "Intensive Care Unit*") in **Title Abstract Keyword**	0

ACM Digital Library	[All: "pressure ulcer∗"] OR [All: bedsore∗] OR [All: "bedsore∗"] OR [All: "decubitus ulcer∗"] OR [All: "pressure sore∗"] OR [All: "pressure injur∗"] **AND** [All: "machine learning"] OR [All: "artificial intelligence"] OR [All: "deeplearning"] OR [All: "supervised machine learning"] OR [All: "unsupervised machine learning"] OR [All: "neural networks"] OR [All: "support vector machine"] OR [All: "machine learning"] OR [All: "artificial intelligence"] OR [All: "deep learning"] OR [All: "supervised machine learning"] OR [All: "unsupervised machine learning"] OR [All: "neural networks"] OR [All: "support vector machine"] **AND** [All: "icu"] OR [All: "critical care"] OR [All: "intensive care"] OR [All: "critical illness"] OR [All: "critically ill patient∗"] OR [All: "intensive care unit∗"]	23
Web of Science Core Colletion	((TS=("Pressure ulcer*" OR Bedsore* OR "Bed Sore*" OR "Decubitus Ulcer*" OR "Pressure Sore*" OR "Pressure Injur*")) **AND** TS=("Machine Learning" OR "Artificial Intelligence" OR "Deep Learning" OR "Supervised Machine Learning" OR "Unsupervised Machine Learning" OR "Neural Networks" OR "Support Vector Machine")) **AND** TS=("ICU" OR "Critical Care" OR "Intensive Care" OR "Critical Illness" OR "Critically Ill Patient*" OR "Intensive Care Unit*")	25
BASE	("Pressure ulcer*" OR Bedsore* OR "Bed Sore*" OR "Decubitus Ulcer*" OR "Pressure Sore*" OR "Pressure Injur*") **AND** ("Machine Learning" OR "Artificial Intelligence" OR "Deep Learning" OR "Supervised Machine Learning" OR "Unsupervised Machine Learning" OR "Neural Networks" OR "Support Vector Machine") **AND** ("ICU" OR "Critical Care" OR "Intensive Care" OR "Critical Illness" OR "Critically Ill Patient*" OR "Intensive Care Unit*")	9
RCAAP	"Pressure ulcer" AND "Artificial Intelligence"	1
	"Pressure ulcer" AND "Machine Learning"	4

*Abbreviations: ACM - Association for Computing Machinery; BASE - Bielefeld Academic Search Engine; CINAHL - Cumulative Index to Nursing and Allied Health Literature; RCAAP - Repositórios Científicos de Acesso Aberto de Portugal Repositórios Científicos de Acesso Aberto de Portugal.
