# Supplementary material for: Pressure Injury Prediction in Intensive Care Units Using Artificial Intelligence: A Scoping Review
Source: Nurs Rep. 2025 Apr 9;15(4):126. doi: 10.3390/nursrep15040126 (PMC12030323; doi:10.3390/nursrep15040126)
Supplement: Supplementary file 1 [file nursrep-15-00126-s001.zip › Supplementary Material S2.pdf]

Supplementary Material S2: Input variables employed in each of the included studies and most significant variables for model training.

Author	Input Variables	Most significant variables for model training
Cho et al. 2013 [32]	**Demographics:** Age, gender; **Administrative data:** Primary diagnosis (ICD-10 code), day count in SICU, nursing units; **Clinical measures and observations:** Systolic BP, ventilator mode, heart rate, body temperature, BMI, APACHE score, consciousness level, incontinence, general edema, degree of edema, number of urinations, number of self-voidings, self-motor response, ostomy, surgical operation, indwelling catheterization, hemodynamic status, other skin lesions, Braden Scale (total score and six subscores); **Nursing Interventions:** Number of position changes, straint, total parenteral nutrition, diet type; **Laboratory Results:** Serum albumin, serum hemoglobin; **Medication:** Number of sedatives, number of analgesics, frequency of medication (including transfusion, IV, and non-IV medication)	-
Kaewprag et al. 2015 [53]	18 medication categories; 61 comorbid conditions; Braden Scale Score	ICD-9: 344, 995, 038, 730, 785, 482, 599, 518, 112, 263; Braden Scale Score.
Kaewprag et al. 2017 [33]	18 medication categories; 61 comorbid conditions; Braden Scale Score	Braden Scale Total Score; Braden Subscale Friction Score; ICD-9: 250, 403, 584, 585, 428, 785, 995, 038, 528, 482, 806, 324, 730, 290.
Alderden et al. 2018 [54]	Delirium; Hypotension; GCS; Oxygenation (SpO2 <90%); Sedation (Riker score); ASA score; Temperature (fever >38ºC); **Vasopressor medication**: Dopamine; Epinephrine; Norepinephrine; Vasopressin; Phenylephrine; BMI at admission; **Laboratory values**: Albumin; Creatinine; Glucose; Hemoglobin; Lactate; Prealbumin; Surgical Time.	BMI; Surgical time; Creatinine; Hemoglobin; Age; Glucose; Lactate; Albumin; Glascow Coma Scale; Hypotension; Prealbumin; Riker Score; CAM; SpO2<90%; Dopamine; ASA score; Norepinephrine; Fever; Epinephrine
Cramer et al. 2019 [55]	**Demographics:** Age; Gender; Weight; Ethnicity; Insurance status; **Admission:** Time from hospital to ICU admission; No prior admission with ulcer; Admitting ICU ward; Stage 1 PU in first 24h; Pressure reduction device; Noninvasive ventilation; Mechanical ventilation; **Physiology:** GCS; Mean BP; Oxygen saturation; Arterial pO2; Arterial pCO2; Hemoglobin; Hematrocit; White blood cell count; Neutrophils; Platelet count; Blood glucose; Sodium; Potassium; Creatinine; BUN; Albumin; Total bilirrubin; Troponin; INR; **Chronic Diseases:** Diabetes; Neuropathy; Peripheral vascular disease; Amputation; Spinal cord injury; Coronary artery disease; Leukemia; Stroke; Heart Failure; Anemia	Stage 1 PI within first 24h; GCS; BUN; Arterial PaO2; Cardiac Surg. Recovery Unit; Albumin; Medical ICU; Hemoglobin; Pressure reduction device; Mean BP.
Hyun et al. 2019 [56]	Age, Gender; Weight; Diabetes; Vasopressor; Isolation; Endotracheal tube; Ventilator episode; Braden Score; Ventilator Days; Length of ICU stay	Age, Gender, Weight, Diabetes, Vasopressor, Isolation, Endotracheal tube, Ventilator episode, Braden score, and ventilator days
Choi et al. 2020 [57]	Bite-block use; Commercial ETT holder use; Duration of ventilator use; Sedative use; Vasopressor use; Steroid use; White blood cells; Hemoglobin; Hematocrit; Albumin.	**Lower mucosal PI:** Bite-block or airway use; Commercial ETT holder use; Corticosteroid use; **Upper mucosal PI:** Commercial ETT holder use; Vasopressor use; Hematocrit; Albumin.

Ladíos-Martin et al. 2020 [21]	Medical service; Days of oral antidiabetic agent or insulin therapy; Ability to eat; Number of red blood cell units transfused; Hemoglobin; PI present on admission; APACHE II scores; Admission diagnosis; Parenteral or enteral nutrition; Ability to control urination; Cardiac drug treatments; Days of cardiac treatment; Mobility type; History of chronic obstructive pulmonary disease; Admission service; Type of activity; Patient age range; Treatment with sedatives or anesthetics; Physical condition; Type of incontinence; History of cancer; History of dementia; History of diabetes	Medical service; Days of oral antidiabetic agent or insulin therapy; Ability to eat; Number of red blood cell units transfused; Hb; PI present on admission; APACHE II scores.
Vyas et al. 2020 [58]	Braden scale subscales: Mobility; Activity; Sensory perception; Skin moisture; Nutritional state; Friction/Shear	-
Alderden et al. 2021 [59]	**Laboratory data:** Serum lactate; Serum Creatinine; Serum Glucose; Hemoglobin; Serum Albumin; Arterial PaO2; Arterial pH. **Nursing skin assessment data:** Thin Epidermis; Skin Tear; Community Acquired Pressure Injury. **Surgical time:** Longest simple surgery. **Vasopressor infusions:** Dose of vasopressin; dose of Norepinephrine infusion; Epinephrine infusion; Phenylephrine infusion; Dopamine infusion; Vasopressin infusion. **Other potential predictors:** MEWS Score; GCS score; Fluid Status; Length of ICU stay prior to HAPI development; Riker score; Admission BMI. **Braden Scale scores:** Braden Total Score; Braden Mobility Subscale; Braden Friction/Shear Score	-
Alderden et al. 2022 [60]	**Demographic and discharge information:** Age, Gender; Race; Ethnicity, hispanic; Hospital length of stay; Died during hospitalization; Time in the emergency department. **Braden Scale scores:** Minimum total score; **Treatments:** Ventilator days; Reintubation; Dialysis; Vasopressor infusion. **Laboratory Values:** Lactate; Serum creatinine; Serum Glucose; Hemoglobin; Albumin; PaO2; PaCO2; pH; **Nursing Skin Assessments:** Fragile skin; Excessively moist skin; Pitting edema. **Nutrition:** unplanned weight loss before admission. **Comorbid conditions and severity of illness:** Charleston Comorbidity Index; MEWS score; Diabetes; Spinal cord injury; Heart Failure; Chronic Obstructive Pulmonary disease	Hemoglobin; Fragile skin; Serum albumin
Šín et al. 2022 [61]	Age; Gender; Ethnicity; ICU Length; input; output; Height; Weight; BP; Glucose; SpO2; Braden SubScales; Albumin; Protein; Bilirubin; Diagnostics: Spinal Injury, Diarrhea, Fracture	Age; Gender; Ethnicity; ICU Length; input; output; Height; Weight; BP; Glucose; SpO2; Braden SubScales; Albumin; Protein; Bilirubin; Diagnostics: Spinal Injury, Diarrhea, Fracture
Ho et al. 2024 [62]	-	-
Kim et al. 2024 [51]	**Demographics:** Age; Gender; Height; Weight; BMI; **Underlying Disease:** Diabetes; Cerebrovascular Disease; Hypertension; Arterial Disease; Skin Disease; **Vital Sign:** Systolic BP; Diastolic BP; Mean BP; Heart Rate; Respiratory Rate; Temperature; SpO2; **Medication:** Relaxant, Sedation, Vasopressor; **Sedation Scale:** SAS. **Laboratory Finding:** arterial pH, PaCO2, PaO2, Bicarbonate, Lactate, BUN, Creatinine, Glucose, Cholesterol, Albumin, Total Bilirubin, AST, ALT, WBC, Hemoglobin; **Braden Scale:** Perception; Moisture; Activity; Mobility; Nutrition; Friction/Shear; **Invasive Procedure**: Invasive Ventilation, CRRT, ECMO; **Others:** Repositioning, Restraint, Diarrhea	Braden-Friction; Respiratory Rate; Diarrhea; Invasive Ventilation; Braden-Perception; Restraint; Braden-Nutrition; Braden-Mobility; Sedation; Braden-Activity

Note: "-" means not reported data. Abbreviations: APACHE – Acute Physiology and Chronic Health Evaluation; ASA – American Society of Anesthesiologists; BMI – Body Mass Index; BP – Blood Pressure; BUN – Blood Urea Nitrogen; CRRT – Continuous Renal Replacement Therapy; ECMO – Extracorporeal Membrane Oxygenation; ETT – Endotracheal Tube; GCS – Glasgow Coma Scale; ICD – International Classification of Diseases; ICU – Intensive Care Unit; MEWS – Modified Early Warning Score; PaCO2 – Partial Pressure of Carbon Dioxide; PaO2 – Partial Pressure of Oxygen; PI – Pressure Injury; SICU – Surgical Intensive Care Unit; SpO2 – Peripheral Oxygen Saturation; WBC – White Blood Count.
