# Supplementary material for: Pressure Injury Prediction in Intensive Care Units Using Artificial Intelligence: A Scoping Review
Source: Nurs Rep. 2025 Apr 9;15(4):126. doi: 10.3390/nursrep15040126 (PMC12030323; doi:10.3390/nursrep15040126)
Supplement: Supplementary file 1 [file nursrep-15-00126-s001.zip › Supplementary Material S3.pdf]

Supplementary Material S3: Performance results of the best model for each study included in the review.

Author	Model	AUROC	AUPR	F1 Score	ACC	SEN	SPE	PPV	NPV	TPR	TNR	FPR	Youden Index	FNR
Cho et al. 2013 [32]	Bayesian Networks	0.85	-	-	-	0.82	0.76	0.36	0.96	-	-	-	-	-
Kaewprag et al. 2015 [53]	Logistic Regression	0.83	-	-	-	0.16	0.99	0.556	0.934	-	-	-	-	-
Kaewprag et al. 2017 [33]	Bayesian Networks	0.827	-	-	-	0.455	0.908	0.292	0.953	-	-	-	-	-
Alderden et al. 2018 [54]	Random Forest	0.79	-	-	-	-	-	-	-	-	-	-	-	-
Cramer et al. 2019 [55]	Logistic Regression	-	-	-	-	0.68	-	0.09	-	-	-	-	-	-
Hyun et al. 2019 [56]	Logistic Regression	0.737	-	-	0.917	0.65	0.693	0.211	0.956	-	-	-	0.342	-
Choi et al. 2020 [57]	Gaussian Naives Bayes	0.82	-	-	-	0.6	0.891	0.231	0.976	-	-	-	-	-
Ladíos-Martin et al. 2020 [21]	Logistic Regression	0.88	-	-	0.87	0.75	0.88	0.2195	0.9868	-	-	-	-	-
Vyas et al. 2020 [58]	XGBoost	-	0.76	-	0.9499	0.8437	0.9726	0.8678	0.9669	-	-	-	-	-
Alderden et al. 2021 [59]	GBM	0.822	-	0.26	-	-	-	-	-	-	-	-	-	-
Alderden et al. 2022 [60]	Ensemble SuperLearner	0.807	-	-	-	-	-	-	-	-	-	-	-	-
Šín et al. 2022 [61]	Random Forest	0.994	-	0.93	0.96	0.916	-	0.946	-	0.92	0.98	0.02	-	0.08
Ho et al. 2024 [62]	MedaBoost	0.908	0.493	-	-	-	-	-	-	-	-	-	-	-
Kim et al. 2024 [51]	GRU-D++	0.945	0.742	-	-	-	-	-	-	-	-	-	-	-

Note: “-“ means not reported data. Abbreviations: ACC – Accuracy; AUPR – Area under the precision recall curve; AUROC - area under the receiver operating characteristic curve; SEN – sensitivity; SPE – specificity; PPV - Positive Predictive Value; NPV - Negative Predictive Value; XGBoost - eXtreme Gradient Boosting Machine; GBM - Gradient Boosting Machine; GRU-D++ - Gated Recurrent Unit with a decay; MedaBoost - Medical Expert Disagreement Adaptative Boosting; TPR – True positive rate; TNR – True negative rate; FPR – False positive rate; FNR – False negative rate.
